# MT-45: A Synthetic Opioid With an Unusual Adverse Effect Profile

**DOI:** 10.7759/cureus.83771

**Published:** 2025-05-09

**Authors:** Markus D Moore, Adil K Shivji, Kerry Anne Rambaran, Steven W Fleming, Saeed K Alzghari

**Affiliations:** 1 Pharmacy, Texas Health Harris Methodist Hospital Fort Worth, Fort Worth, USA; 2 Emergency Medicine, University of California San Diego - East Campus Medical Center, San Diego, USA; 3 Toxicology, North Louisiana Crime Laboratory, Shreveport, USA; 4 Louisiana Addiction Research Center, Louisiana State University Health Sciences Center, Shreveport, USA

**Keywords:** adverse effects, drug of abuse, mt-45, opioids, synthetic opioids

## Abstract

The opioid crisis continues to be a problem in the United States. The rise of illicit synthetic opioids continues to be a threat to those that may unknowingly digest these substances. Herein, we present a synthetic opioid, MT-45, that has an unusual adverse effect profile. In one case series, three patients experienced ototoxicity. In a separate case series, three patients experienced a range of symptoms that included visual impairment, dry eye disorder, dermatitis, Mee's lines, alopecia, and hair depigmentation. Clinicians should consider MT-45 as part of their differential diagnosis if a patient is experiencing an uncommon adverse effect associated with MT-45.

## Editorial

Opioids are a class of drug that binds to opioid receptors throughout the brain and body, originally synthesized for analgesic use [1]. Common effects associated with opioid use include euphoria and sedation; in turn, these effects put people at risk for the overuse of opioids that has led to the current opioid crisis. The Centers for Disease Control and Prevention (CDC) estimated there were 81,806 opioid overdose deaths in 2022, of which 90% involved synthetic opioids other than methadone [2].

MT-45, also known as 1-cyclohexyl-4-(1,2-diphenylethyl)piperazine, is a synthetic opioid analgesic that is used recreationally. Like other opioids, MT-45 users experience side effects such as respiratory depression, decreased level of consciousness, and withdrawal syndrome [3]. What makes this synthetic opioid unique is its other complications that can cause ototoxicity, visual impairment, dry eye disorder, dermatitis, Mee's lines, alopecia, and hair depigmentation [4-5]. This adverse effect profile is specific to MT-45, and providers in the emergency room or clinic settings can utilize this information along with an opioid toxidrome to assist them in their clinical diagnosis.

In a case series of nine patients who experienced MT-45 intoxication, three of them experienced various ototoxic symptoms that included some degree of hearing impairment (Table 1) [4]. All three individuals were males in their late twenties. Two patients experienced reversible ototoxic damage; however, the third patient had a follow-up audiology test two weeks afterwards, and it was concluded that the effect of the MT-45 had likely caused permanent hearing impairment. Of the three that were affected, one individual had 3-methoxyphencycylidine, a hallucinogen related to phencyclidine (PCP), detected in their urine. While the effects of 3-methoxyphencycylidine may have played a role in the individual’s clinical symptoms, this patient only experienced temporary hearing loss. All patients who experienced ototoxic effects were initially treated with naloxone, a pure opioid antagonist, with total doses ranging from 0.4-0.8 mg.

**Table 1:** Clinical presentation of MT-45 users that experienced auditory effects

Case	Clinical symptoms	Substances detected in urine sample (excluding MT-45)	Reported route of MT-45 administration
Case 1 [4]	Unconsciousness, apnea, cyanosis, miosis, hearing impairment	3-methoxy-phencyclidine	Nasal
Case 2 [4]	Unconsciousness, respiratory depression, hand weakness, hearing impairment	None	Information not given
Case 3 [4]	Unconsciousness, apnea, miosis, ventricular dysfunction, respiratory depression, hearing loss	None	By mouth or intravenously

In a different case series, a wide array of symptoms was observed in three patients, ranging from widespread rashes, hair depigmentation or loss, white discoloration of the nails, and various ophthalmic complications (Table 2) [5]. All patients in this case series were also males; however, the age range was from their mid-twenties to mid-thirties. All three cases involved patients with a pronounced spread pattern rash. The symptoms were described as follicular-based papules and pustules with various areas of scaly dermatitis that were more painful than itchy for the affected patients. The dermatological conditions did seem to resolve gradually with no permanent side effects. Regarding the hair loss found in all three cases, all patients had their hair grow back in some capacity. Each patient’s hair was discolored and would not return to its original color until several months later. Mees’ lines were also present in two of the cases. Due to these symptoms, the clinicians presumed thallium poisoning; however, thallium was not detected in their system. Lastly, all three cases had some form of ophthalmic symptoms. One patient did not suffer from vision loss, but the other two patients had to undergo cataract surgery to correct their vision loss.

**Table 2:** Dermatological and ocular adverse effects of MT-45 users

Case	Skin	Hair/Nail	Eye
Case 1 [5]	Rash present	Hair loss, depigmentation of eyebrows and eyelashes only	Dryness, vision loss
Case 2 [5]	Rash present	Hair loss, depigmentation of scalp, eyebrows, and eyelashes. Mees’ lines present	Conjunctivitis, vision loss
Case 3 [5]	Rash present	Hair loss, depigmentation of scalp, eyebrows, and eyelashes. Mees’ lines present	Redness, dryness

Both studies showcase the range of symptoms caused by MT-45. Highlighting the two studies showcases the toxic effects of this drug on one’s hearing, sight, skin, and hair. Clinicians should be aware of these specific and unusual adverse effects of MT-45. While we do not know the full range of acute and chronic complications from MT-45, we aim to provide a clearer understanding of the side effect profile of this opioid. We hope health care providers can include this synthetic opioid as part of their differential diagnosis and pair it with a patient’s clinical presentation.
